# Wild cricket social networks show stability across generations

**DOI:** 10.1186/s12862-016-0726-9

**Published:** 2016-07-27

**Authors:** David N. Fisher, Rolando Rodríguez-Muñoz, Tom Tregenza

**Affiliations:** 1 Centre for Ecology and Conservation, Penryn Campus, University of Exeter, Penryn, TR109FE Cornwall UK; 2 Department for Integrative Biology, Summerlee Science Complex, University of Guelph, Guelph, N1G 2W1 ON Canada

**Keywords:** Exponential random graph models, Gryllus, Network comparison, Population structure

## Abstract

**Background:**

A central part of an animal's environment is its interactions with conspecifics. There has been growing interest in the potential to capture these interactions in the form of a social network. Such networks can then be used to examine how relationships among individuals affect ecological and evolutionary processes. However, in the context of selection and evolution, the utility of this approach relies on social network structures persisting across generations. This is an assumption that has been difficult to test because networks spanning multiple generations have not been available. We constructed social networks for six annual generations over a period of eight years for a wild population of the cricket *Gryllus campestris*.

**Results:**

Through the use of exponential random graph models (ERGMs), we found that the networks in any given year were able to predict the structure of networks in other years for some network characteristics. The capacity of a network model of any given year to predict the networks of other years did not depend on how far apart those other years were in time. Instead, the capacity of a network model to predict the structure of a network in another year depended on the similarity in population size between those years.

**Conclusions:**

Our results indicate that cricket social network structure resists the turnover of individuals and is stable across generations. This would allow evolutionary processes that rely on network structure to take place. The influence of network size may indicate that scaling up findings on social behaviour from small populations to larger ones will be difficult. Our study also illustrates the utility of ERGMs for comparing networks, a task for which an effective approach has been elusive.

**Electronic supplementary material:**

The online version of this article (doi:10.1186/s12862-016-0726-9) contains supplementary material, which is available to authorized users.

## Background

Alongside elements of their environment such as climate, resource availability and predation risk, animals are also adapted to their social environment. This is comprised of the social interactions with con-specifics, through mating, fighting, playing, grooming or associating in the same group. If the organism has some choice over its social interactions, it will partially construct the social environment it experiences. This social environment can be characterised as a social network, where individuals (“nodes”) are connected with others that they interact or associate with via links called “edges” [1]. Having been adopted from the study of human social behaviour, the study of animal social networks is now out of its infancy, with studies across a range of taxa and addressing a wealth of different questions [2–6].

Studies on animal social networks typically construct a social network from a single continuous period of observation. This allows one to make conclusions about ecological processes over the time period that relate the social environment to other aspects of the animals’ ecology, for example their exposure to disease [7–9] or group decision making [10, 11]. Studies on networks rarely extend to timescales that would allow evolutionary processes. This is probably because most animals studied through social network analysis are relatively long-lived vertebrates e.g. dolphins, baboons or great tits. Studies over multiple generations therefore take decades, and so are uncommon [12]. This is problematic, as we do not know the extent to which the characteristics of the social network structure of populations persist across generations. Qualitatively similar processes predicting the structure of social networks have been found in sperm whales (*Physeter macrocephalus*) [13] long-tailed manakins (*Chiroxiphia linearis*) [14] and spotted hyenas (*Crocuta crocuta*) [15] in different years, but none of these studies spanned multiple generations. Shizuka et al. [16] demonstrated that distinct communities of golden-crowned sparrows (*Zonotrichia atricapilla*) persisted across three seasons, despite high turnover of individuals. However, two generations at most may have featured in this study, limiting conclusions relating to stability across evolution time. In captive rhesus monkeys (*Macaca mulatta*), the mother’s social network has shown to be a good predictor of the daughter’s social network [17]. Furthermore, Brent et al. [18] showed that there is a heritable component to social behaviour in a population of free living rhesus monkeys. Both of these indicate there should be consistency of the local social network across generations, but whether this is true for the population’s social network has not been investigated.

If social network structure does not resist the turnover of individuals, then evolutionary processes facilitated by the presence of a social network may not actually occur. For example, the evolution of cooperation is facilitated by a viscous social network, allowing co-operators to preferentially interact with each other and avoid cheats [19–21]. However, if the structure of the network changes from generation to generation, then a cooperative strategy that exploits aspects of the social network in one generation might not be successful in the next, preventing it from persisting in the population. Evolutionary processes and responses such as this cannot take place if the social network structure is unstable, in the same way that animals cannot evolve a particular thermal tolerance if the temperature of their environment fluctuates randomly over generations. Furthermore, it is currently debated whether niche construction occurs in a systematic manner [22, 23]. By regularly measuring the social niches that individuals construct in multiple independent generations, we can determine whether a similar social environment is constructed each time (evidence of a systematic process) or if the social environment differs from year to year (no evidence for a systematic process).

We wanted to determine whether a population showed consistent social network structure across independent generations by studying a species with non-overlapping generations. Independent generations are necessary, as one keystone or despotic individual could have a large influence on network structure over time if they were long-lived [24–26]. We assessed whether the factors predicting the structure of social networks in a population of wild field crickets (*Gryllus campestris*) were consistent across years by using model parameters based on networks in one year to simulate networks from other years. If networks from a year could be used to accurately simulate the characteristics of networks from others, it would indicate that social network structure is conserved over time. We also related the ability of one network to predict another with the difference in time (years) between them and the difference in total population size between them.

## Methods

### Study system & data collection

The field cricket *G. campestris* is a univoltine species, with non-overlapping generations. Adults emerge early in spring having overwintered as nymphs in a burrow they dug themselves in the autumn (burrows do not persist across generations), and are active from April-July. Once sexually mature, adult males start singing to attract females, and both sexes move around burrows to find mates [27]. Females tend to move more than males [28], but both sexes spend some time guarding a burrow and some time moving between different burrows. They will also fight members of the same sex for access to burrows or mating partners [29], although we do occasionally observe aggressive interactions between the sexes (pers. obs.). This allows us to construct social networks between individuals that either mate with or fight each other.

Our study site is a meadow of approximately 20 by 40 m, located on a north facing slope in a valley in Northern Spain. We have been studying *G. campestris* there since 2005, with a generation each year. Such timescales are long enough to allow contemporary evolution [30], with adaptations with major implications for fitness able to occur in only one generation [31]. Once nymphs become active after overwintering, we located each burrow at our study site and marked it with a unique number. We placed video cameras over burrows with an active individual before any adult emergences were observed. Cameras recorded cricket activity 24 h a day, seven days a week using infrared illumination at night. Nymphs rarely move among burrows (Rodríguez-Muñoz, pers. obs.). Therefore, the camera footage along with direct observations of burrows without cameras allowed us to determine when each individual became an adult. Two-three days after it emerged as an adult, we caught each cricket and fixed a unique waterproof vinyl tag to its thorax with cyanoacrylate glue. This allows non-invasive identification of individuals recorded on the video. Following this, we released crickets back to the burrow we caught them from. Crickets use burrows to hide from predators such as robins and shrews, and spend most of their life in the immediate vicinity of burrows, usually within the frame of our cameras. They will share burrows with members of the opposite sex while mating with them, but tend to fight members of the same sex when they approach. Therefore, the vast majority of cricket social interactions take place at burrows, and so are recorded by our cameras. If we did not directly record the death of a cricket we set it as the day after we last observed it. Migration in and out of our population is limited by surrounding unsuitable habitat [32], so we are confident that the majority of crickets active in the population are caught and tagged. Of the years since 2005, we have completely analysed the video from 2006–08 & 2011–13, so we present those six years in this study.

Social interactions are either fighting, which typically only occurs within the sexes, and mating. Here we present social networks based on both types of interactions, so that all individuals could theoretically interact with each other. We directly record interactions rather than infer associations, defining an edge if two individuals ever mated or fought, and setting the edge weight as the number of interactions between them. This gives weighted, symmetrical (undirected) networks.

### Exponential random graph models

We used exponential random graph models (ERGMs, also known as p* models) to quantify the networks’ properties [33]. These have previously been used in animal behaviour research to investigate the structure of dominance hierarchies in pukeko (*Porphyrio melanotus melanotus*) [34], and the structure and stability through time of cooperative leks in male long-tailed manakin (*Chiroxiphia linearis*) [14]. ERGMs are similar to logistic regression models and have been developed to model the presence and strength of edges in a network [35–38]. This makes it possible to determine which variables contribute to non-random network structure, which can provide insights into the social processes forming the network [39, 40]. Variables predicting edge formation and strength can be structural properties of the network (for instance the presence of a mutual association creating an association between two individuals: “triadic closure”), properties of the individuals (for instance their sex), or properties of a relationship between two individuals (for instance their genetic relatedness). Which predictor variables are chosen depends on the interests of the researcher and the available data, as for a regression [39]. Effect sizes for each variable are arrived at through a stochastic process of model fitting. These effect sizes can be transformed to probabilities, allowing the influence of variables to be interpreted in their own units and so facilitating the comparison of effects [37]. Importantly, by estimating multiple different processes in one model, each term is calculated relative to the others, and so shared influence on edge formation is accounted for. For instance, we can model the effect of spatial distance between a pair on edge formation, and then quantify the effect of other, more explicitly social processes beyond the influence of space. Once coefficients for each variable have been estimated, these can be used to simulate a range of new, otherwise random networks to compare with the original network [37, 38]. Furthermore, coefficients from one model can be applied to simulations based on a different network. This allows one to determine how well the parameters predicting one network predict the observed structure in other networks.

### Efficacy of network simulation

We first determined how well fully parameterised models simulated various network metrics compared with much reduced models. This would tell us whether our models were effective at simulating realistic networks. For the network in each year we fitted an ERGM with the same effects using the packages “ergm” [37] and “ergm.count” [41] in R [42]. The effects in this full model were:

- Conway-Maxwell-Poisson (CMP) distribution. This models the tendency for the distribution of edge weights to be under- or over-dispersed relative to a theoretical Poisson distribution, analogous to a quasi-Poisson parameter in a glm [41].
- Non-zero. This models the tendency for networks to be sparse e.g. individuals are not connected to every other individual in the network. This is a common attribute of social networks [43].
- Transitive ties. This models triadic closure, the tendency for crickets to interact with those with whom they share a mutual 3^rd^ interaction. This is a common property of social networks [2].
- Main effect of sex. This models any sex differences in total interaction strength, summed across all interactions. Both sexes are promiscuous [27] and males cannot control access to females [44] so we do not expect major sex differences in interaction frequency. Females are modelled as the default with males as the contrast.
- Node-matching by sex. This models the tendency for crickets to interact more or less with individuals of the same sex as themselves. As matings (inter-sex; 4311 recorded in total in 2006–08 & 2011–13) are more common that fights (typically intra-sex; 1628 recorded in total in 2006–08 & 2011–13), we expect this to be negative.
- Emergence location closeness. This dyadic covariate contains information on the closeness (the inverse of distance) between the adult emergence co-ordinates of each pair of crickets. We expect this variable to be positive, as individuals emerging closer together should interact more.

If the initial run of a model did not achieve convergence (as indicated by the ergm.count package) we then re-ran the model, using the estimated coefficients of each parameter as new starting values for the next run [37] in a similar manner to that advocated for Stochastic actor-orientated models [45]. This either lead to satisfactory convergence or only made small differences to the coefficients, indicating the parameter values were relatively stable and thus were reliable. We then simulated 100 new networks based on all the coefficients from the model, and 100 new networks using only the CMP and non-zero parameter coefficients. Comparison of these two sets of 100 networks for each year would indicate how effective our model was at reproducing elements of the real cricket social network. The elements we chose were the mean path length (or geodesic distance) of the network, the degree correlation of the network, and the clustering coefficient. The mean path length is the average number of steps (edges) on the shortest route between all possible pairs of individuals [46]. Individuals that are separated from each other completely are recorded as having an infinite distance between them, and these path lengths were not used in the analysis. The degree correlation is the correlation between the degree (the number of unique connections) of the individuals at either end of each edge [47]. The clustering coefficient is the ratio of open triads (where two crickets are connected to a third but not to each other) to closed triads (where all three are connected) and is a measure of local edge density [48]. In theory, any network metric could be used, we chose these as they are commonly used and represent features of the network with global implications based on local connections. We then calculated “predictive distances” for each year and for each network metric. These were simply the difference between each of the 100 simulated values and the real value for each network metric, for each year, for both the simulations with all parameters and the simulations with the reduced parameters. We then compared the absolute size of these using Wilcoxon rank sum tests, to determine whether the simulation with all terms gave significantly shorter predictive distances than the reduced-term simulations.

### Within- and between-year simulation efficacy

The above analysis looks at capacity for an ERGM to simulate a network based on a model from the same year, hereafter a within-year comparison. We also wished to determine whether ERGMs from the other years could accurately simulate a network in a different year, a between-year comparison. If they could, we would have evidence of similarity, and so stability, of network characteristics across years.

We took the model parameter coefficients from the full model for each year, and used them to simulate 100 new networks from each other year. We entered the original network and its exact characteristics (population size, sex ratio, total number of interactions and emergence location of individuals) into these simulations, so the simulations were as realistic as possible. We then calculated predictive distances as before for each set of simulations. Therefore, alongside the 100 predictive distances for the model in 2006 predicting the clustering coefficient in 2006 (within-year comparison), we had 100 predictive distances for the model in 2006 predicting the clustering coefficient in 2007 (between-year comparison), and so on. We then took the mean of each of these set of 100 values and compared the between-year comparisons with the within-year comparisons using Wilcoxon rank sum tests.

### Predictive distances and other population characteristics

We compared the sizes of the mean predictive distances between years to the difference in time (number of years) and differences in population size (number of individuals) between those years. For this we used Mantel tests [49] in the package vegan [50] to account for the fact that we compared each year to multiple others, who were also involved in multiple comparisons, like a network. We calculated a Spearman’s rank correlation coefficient as the distribution of values was non-normal. A positive relationship between distance in time and predictive distance would indicate that the networks were changing over time, weakening the relationships among them. No relationship would be taken as further evidence of network stability across generations. Network size is an important axis of variation, so networks that are more different in size may be worse at predicting each other. In which case we expect a positive relationship between predictive distance and difference in population size.

## Results

### Predictors of cricket social networks

The variable estimates for each year are shown in Table 1. In general, the CMP parameters are positive, indicating over-dispersion, and the non-zero parameters are negative, indicating that most possible edges did not exist/were zero i.e. crickets tended not to be connected to all others. The transitive ties parameters were positive, indicating that the presence of a mutual connection increased the likelihood that two crickets would interact. The main effect of sex was generally weak and negative with relatively large standard errors, indicating only a weak tendency for males to interact slightly less often than females. The node-matching by sex was negative, indicating that intersexual interactions were more common than intrasexual interactions. The dyadic effect of emergence location was positive, confirming that individuals emerging close together interacted more. Each effect is estimated while accounting for the other effects, so the process of triadic closure is significant even given that crickets emerging near each other are more likely to interact.

**Table 1:** Parameter estimates from ERGMs in each year, with standard errors in brackets. CMP stands for Conway-Maxwell-Poisson distribution; see main text for description of terms

Parameter	2006	2007	2008	2011	2012	2013
CMP	0.923 (0.013)	0.783 (0.010)	0.914 (0.058)	0.743 (0.012)	0.958 (0.012)	0.600 (0.082)
Non-zero	−7.109 (0.436)	−6.389 (0.192)	−4.287 (0.282)	−6.622 (0.231)	−5.805 (0.476)	−6.377 (0.176)
Transitive ties	1.954 (0.208)	1.373 (0.090)	0.654 (0.136)	1.571 (0.113)	1.508 (0.230)	1.323 (0.077)
Sex	−0.096 (0.024)	0.059 (0.017)	−0.092 (0.109)	−0.019 (0.015)	−0.107 (0.028)	−0.073 (0.042)
Node-match by sex	−0.411 (0.030)	−0.399 (0.036)	−0.685 (0.162)	−0.339 (0.023)	−0.430 (0.035)	−0.020 (0.081)
Emergence location	0.390 (0.036)	0.391 (0.066)	0.269 (0.113)	0.594 (0.0425)	0.143 (0.055)	0.679 (0.199)

### Full vs. reduced simulations

For mean path length, the full simulations gave significantly smaller predictive distances than the reduced simulations in all years apart from 2012, when the full simulations actually gave larger predictive distances (all Wilcoxon rank sum tests, *p* ≤ 0.001 in all cases).

For degree correlation the reduced simulations gave smaller predictive distances in all years (all Wilcoxon rank sum tests, *p* ≤ 0.038 in all cases) except 2012, where the difference was not significant (Wilcoxon rank sum test, *p* = 0.080) and in 2013, where the full simulations gave significantly shorter predictive distances (Wilcoxon rank sum test, *p* < 0.001).

For clustering coefficient the full simulations gave significantly smaller predictive distances in all years (all Wilcoxon rank sum tests, *p* < 0.001) except 2008, where the full and reduced models gave equal predictive distances (Wilcoxon rank sum test, *p* = 0.085). Box plots for all these comparisons are shown in the Additional file 1: (Fig. S1-3).

From these results we concluded that our models were effective for predicting path lengths and clustering coefficients, but not degree correlations. Therefore, we did not consider degree correlations for the rest of the analyses.

### Predictive distance within vs. between years

**Fig. 1:**
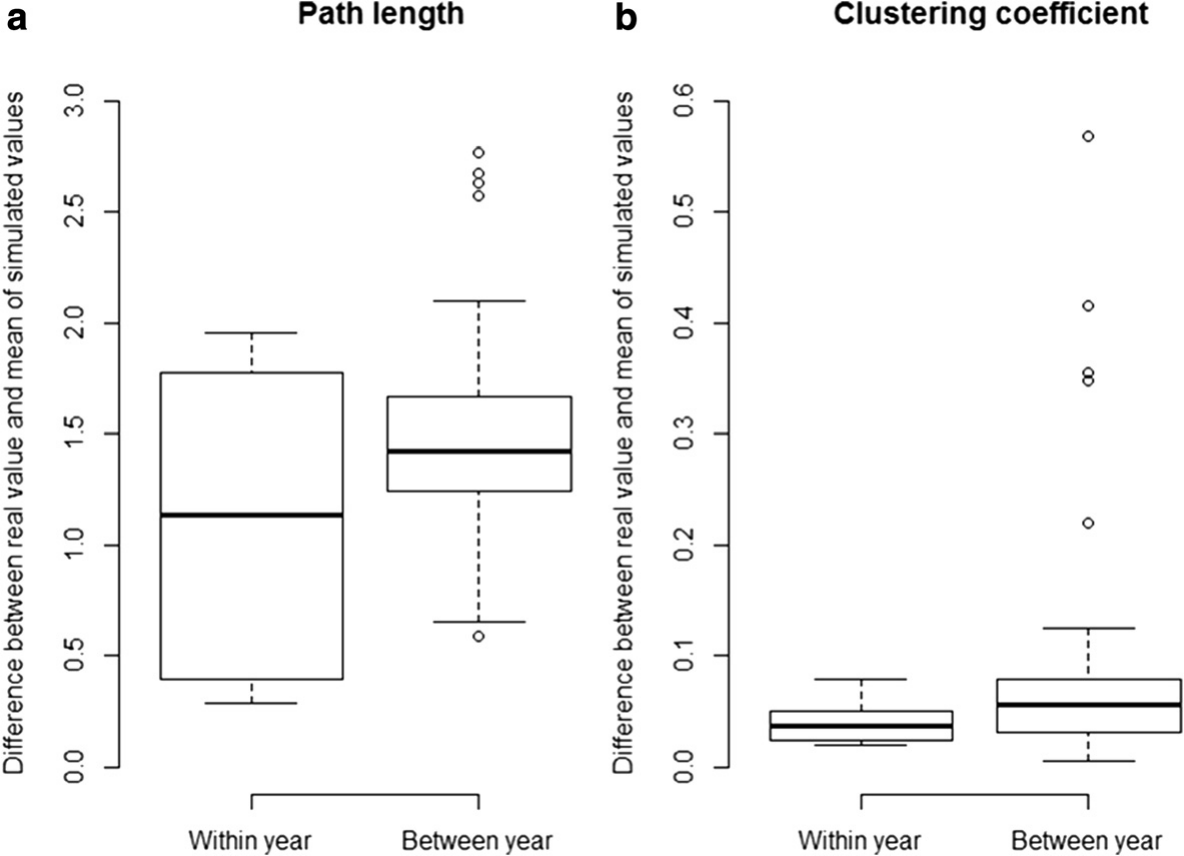
Box plots of the predictive distances for the within- and between-year comparisons for path lengths (**a**) and clustering coefficient (**b**). The y-axis indicates the differences between the observed and simulated network measures. Network metrics are able to predict the true network both within and between years; for both network measures the difference between the within-year and between-year comparison was non-significant (see results)

The predictive distances for the within- and between-year comparisons are shown in Fig. 1a. (path length) & b. (clustering coefficient). For both path length and clustering coefficient the within-year comparisons gave equal predictive distances to the between-year comparison (Wilcoxon rank-sum tests, path length: W = 70, n (within-year) = 6, n (between-year) = 30, *p* = 0.418; clustering coefficient: W = 61, n (within-year) = 6, n (between-year) = 30, *p* = 0.233).

### Correlates with predictive distance

**Fig. 2:**
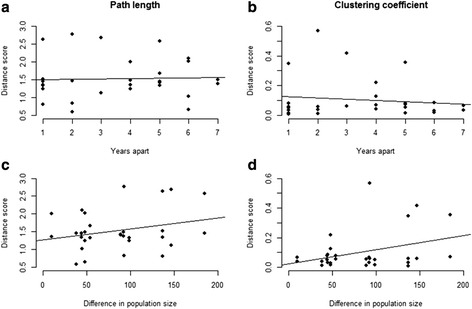
Plots of the predictive distance for the between year comparisons against the difference in time between each pair of compared years (**a** & **b**) and the difference in population size between each pair of compared networks (**c** & **d**). Plots (**a** & **c**) show this relationship for predicted path lengths, (**b** & **d**) for predicted clustering coefficient. Plotted are the means of the 100 predictive distances for each comparison: the difference between the mean of the simulated values and the real value. Distance in years did not affect the ability of models to predict other networks (no correlation: Mantel test, rho = −0.169 & -0.107), but were worse at predicting the path lengths of other networks when they were initially parametrised on networks with different population size (increased predictive distance with increased difference in population size: Mantel test, rho = 0.481 & 0.488) (see results). The lines are from simple regressions of the variable on the x axis on the predictive distance, which are not informed by the Mantel tests but help visualise the result

There was no significant relationship between number of years apart and predictive distance for either path length (Fig. 2a; Mantel test, rho = −0.169, *p* = 0.733) or clustering coefficient (Fig. 2b; Mantel test, rho = −0.107, *p* = 0.708). There were positive, albeit marginally non-significant relationships between difference in population size and predictive distance for path length (Fig. 2c; Mantel test, rho = 0.481, *p* = 0.056) and clustering coefficient (Fig. 2d; Mantel test, rho = 0.488, *p* = 0.060).

## Discussion

### Predictors of cricket social networks

We found that cricket networks were sparse, like most social networks, and the interaction strengths were over-dispersed, suggesting fewer weak interactions and more strong interactions than expected under a Poisson distribution. This may be evidence of preferred associations, with crickets avoiding most individuals to interact strongly with particular others. Consistent mate-choice by females has been shown in captivity in various species [51–54], and individual male traits such as singing frequency and body mass influence mating success in this species [27], so for mating interactions this seems plausible in this system. Male crickets that are in sperm competition are also more likely to fight [55], so crickets may have consistent fighting opponents as well. Crickets interacted more strongly with those that emerged near to them, which was expected, and illustrates the importance of accounting for spatial factors in species whose interactions are likely to be strongly spatially-structured.

We also found that males interacted slightly less often than females, although the reverse was true in 2007 and the standard errors tended to be relatively large. In this polyandrous species both sexes benefit from multiple mating and show highly skewed reproductive success [27] and females may compete strongly with other females to maintain access to the safety of burrows or to prevent sperm-limitation [56, 57]. Therefore, it is not surprising that there are only small or no differences between the sexes in the rate of mating and fighting. The sex-matching parameter was negative in all years, which was expected as mating is more common than fighting, but simulating this helps create more realistic networks. As fighting between a pair of males is related to increased sperm competition between the pair [55] fighting may not be an effective behaviour for avoiding post-copulatory competition, and along with potential costs of injury may explain why it is not more common.

Path length and clustering coefficients were generally simulated more effectively by the full simulations than the reduced simulations. The exception was 2012, for which the full model was not better at simulating clustering coefficients or path lengths. Exactly what was different about 2012 is unclear. Our models were however not effective at predicting degree correlations. Accurately predicting degree correlations in social networks based on randomisations is recognised as difficult [58] hence this is not necessarily as failing unique to ERGMs. We have found that mating networks show positive degree correlations [55], yet most random networks show null or negative degree correlations [59]. This indicates there is some aspect of cricket behaviour that our ERGMs did not capture, such as positive assortment by some trait or aspect of “quality”.

### Stability of networks across generations

The coefficients of each model were largely consistent in size and sign each year, and the predictive power of the ERGMs was equal for within- and between-year comparisons for both network metrics considered. This indicates that networks were comparable between years. We also found no influence of number of years apart on predictive distance between networks. Therefore, the fundamental properties of cricket social networks that we captured do not appear to diverge over time. Together, these results provide strong support for the idea that some of the characteristics of cricket social networks are stable across generations. This would allow the population to adapt to the social environment in the form of the social network structure. If crickets are adapted to this particular social network structure, then artificially altering the network should lead to a reduction in cricket fitness. Manipulations are relatively rare for studies on the social networks of animals [6], but by altering the rate of interactions crickets experience with artificial barriers we could investigate this idea. Furthermore, if networks are stable over evolutionary time, evolutionary processes such as the evolution of cooperation through directed reciprocity could occur [19–21]. This is a key assumption of these models of cooperation and of models of selection acting via social networks [60, 61]. The only direct evidence for cooperation in our species is when males and females share a burrow [44]. Our point is that, for the first time, we have shown that social network structure in the wild is relatively stable across generations, resisting the regular turnover of individuals. This is necessary before any kind of evolutionary processes can take place across networks.

Our results also indicate that the social niche construction undertaken by crickets is a systematic process, with similar result each year. It has previously been debated to what extent niche construction is a systematic process, and whether it typically tends to bring advantages or disadvantages to the organisms that carry it out [22, 23]. Although we have not shown whether the social niche a cricket constructs is beneficial or costly, we have shown the first part of the conjecture to be true for the social environment of field crickets. Niche construction has been predicted to be beneficial more often than not [23], but it is possible that limitations imposed by space use, the use of burrows or the threat of predators leads to crickets constructing disadvantageous social niches. Further work on the growth rate, survivability or reproductive success of crickets with different social niches will help us investigate this.

### Predictive distance increases with difference in population size

We found positive relationships between the predictive distance between years and the difference in population size between those years. These were marginally non-significant in both years treated independently, but as the Mantel test is regarded as overly conservative [62] and since we found the same pattern for both metrics, we are confident that the predictive distances do increase as the population sizes diverge. This is despite the fact that we entered the exact properties of the population for these simulations. This therefore indicates that the network changes in some unexpected way as it changes in size, as otherwise the larger networks would simply scale up accurately from the smaller networks. Similar results have been found across social networks of various species, where larger networks also tended to be more modular than smaller networks, which may limit disease transmission [63, 64]. For the crickets, a possible cause of the change in structure is that the rates of mating and fighting change differently as the network grows. From Additional file 2: Table S1 there appears to be a trend for larger network to have relatively more fights than smaller networks (percentage of all interactions that were fights is 12, 22, 18, 34, 23 and 33 for population sizes 79, 110, 161, 198, 208 and 239 respectively, which gives a correlation of 0.81). Therefore, larger network may be more antagonistic than smaller networks. For the same reason, the node-match by sex effect varied between years. Crickets therefore seem to have different social behaviour depending on the number of potential rivals of the same sex. In the sister species *G. bimaculatus*, males that had been housed with multiple rival crickets exhibited more aggressive song than those housed alone [65]. Furthermore, *G. integer* males produced song less and searched more at high densities [66]. Plasticity such as this could cause individuals’ social environments to vary across a range of densities as we see here.

As a general rule it is not surprising that network similarity is based on size; network size is an important axis of variation. What this suggests is that studies on the social behaviour of small populations, say in captivity or at times of year when individuals live in smaller groups, may not be easily scaled up to situations where the animal lives in larger groups. Many studies on social behaviour in captivity have the express aim of understanding the implications of behaviour for ecological processes such as information or disease transmission in the wild (e.g. [67, 68]). Our findings indicate a need for caution in attempting to transfer this research between contexts.

### Using ERGMs to investigate and compare networks

We have used ERGMs to explicitly compare different networks. The effective comparison of networks of different sizes, from different populations of one species, or across species has long been the subject of study and debate [69, 70]. However, as highlighted by a recent review: “Comparing networks across contexts (e.g. between populations or species) remains one of the main challenges in network analysis” [6]. Part of this challenge is related to differences in data collection among different systems [6]. Yet this has clearly not stopped comparative studies in other fields. Our suggestion is that, as we have demonstrated here, ERGMs can be used to predict the structure of the network of one species or population from the parameters of another. This will likely reveal a range of networks that are successfully able to predict each other, and a range that cannot. Comparison of similar and dissimilar factors between these different networks, e.g. differences in data collection method vs. differences in population size vs. differences in taxonomic group, will then allow us to determine specifically what makes one observed network different or similar to another. Once we understand how factors such as the method of data collection influence the parameter estimates of an ERGM, we can then account for it to explore more interesting questions, such as the phylogenetic conservation of complex social behaviours [71].

## Conclusions

Overall, we found stability in some social networks across generations, and consistency in factors affecting social network structure. This would allow the cricket population to evolve in response to social network structure. It also suggests that crickets construct social niches in a systematic way, although whether this is adaptive or not remains an open question. Alongside our study spanning eight generations, the existence of other studies with long-term data sets of social behaviour in populations [72–75], should mean that soon we should be able to actually detect evolutionary changes occurring in response to variation in social structure. However, our observation that networks more different in size were worse at predicting one-another indicates that social structure may not be consistent between contexts where population sizes differ, such as across seasons or between captivity and the wild. Alongside Edelman and McDonald [14], we have confirmed that ERGMs are a reproducible method for some network metrics by arriving at similar results (size and sign of coefficients) in different years. We have also demonstrated that ERGMs can be used to compare networks distinct in time, and would encourage other researchers to use ERGMs as an effective tool for investigations into network structure and comparisons.

## Additional files

Additional file 1: Fig. S1-3. Plots of the full (left box in each panel) and reduced (right box in each panel) simulations and their predictive distances (y axis). The predictive distance is the difference between the simulated values and the real value from the network. S1 is for mean path length, S2 for degree correlation and S3 for clustering coefficient. See Methods (in the main text) for details on how these were calculated and see Results for which comparisons are statistically significant. (ZIP 21 kb) Additional file 2: Table S1. Characteristics of the cricket population and network statistics for each year. Isolated nodes are individuals that were not recorded to mate or fight another individual. Clustering is the ratio of complete triangles, where three crickets all interact with each other, to all possible triangles, including those where only two of the three crickets actually interact. Betweenness is the number of shortest paths between each pair of crickets in the network that pass through a particular cricket. We have taken the mean across all individuals, giving one measure of how well connected different parts of the network are (high values indicate poorly connected). Path length is the number of edges one must move along to connect any two nodes in a network. We have taken the mean, giving one value for how densely connected the network is (high values indicate low connectivity). (DOCX 16 kb)
