# Supplementary figures and images for: Wild cricket social networks show stability across generations
Source: BMC Evol Biol. 2016 Jul 27;16:151. doi: 10.1186/s12862-016-0726-9 (PMC4964091; doi:10.1186/s12862-016-0726-9)

**2006**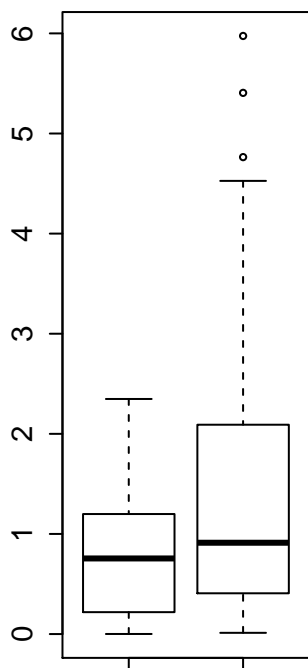**2007**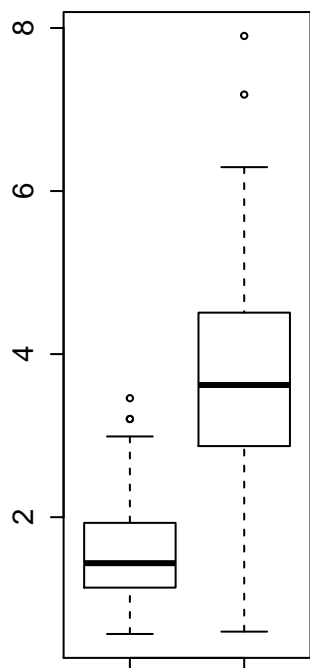**2008**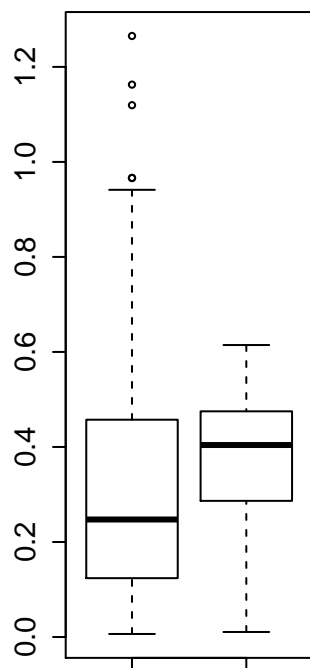**2011**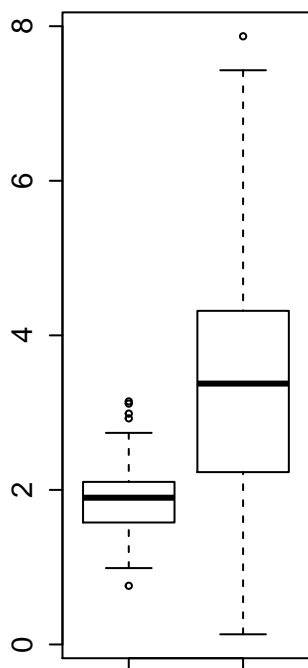**2012**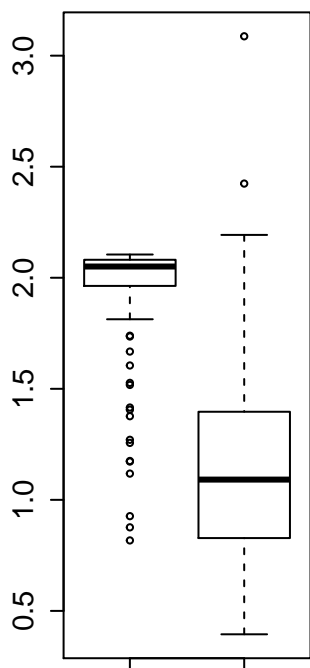**2013**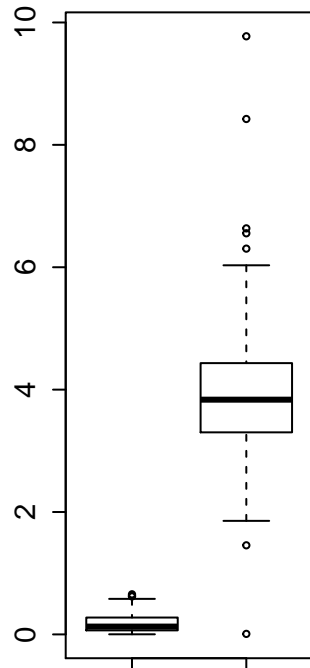

Supplement: Additional file 1: Fig. S1-3. — Plots of the full (left box in each panel) and reduced (right box in each panel) simulations and their predictive distances (y axis). The predictive distance is the difference between the simulated values and the real value from the network. S1 is for mean path length, S2 for degree correlation and S3 for clustering coefficient. See Methods (in the main text) for details on how these were calculated and see Results for which comparisons are statistically significant. (ZIP 21 kb) [file 12862_2016_726_MOESM1_ESM.zip › SN across years figS1 PL.pdf]

**2006**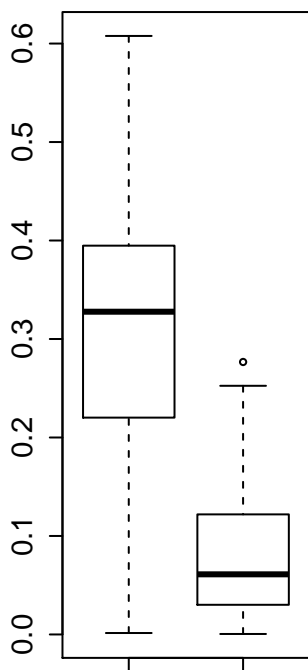**2007**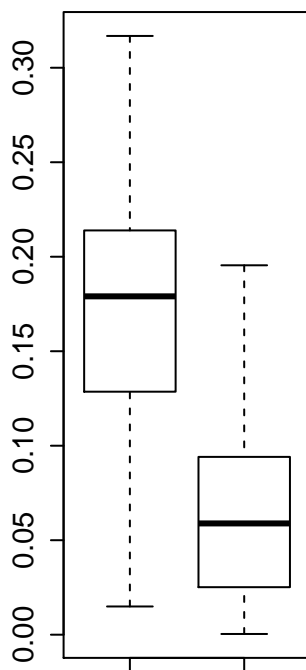**2008**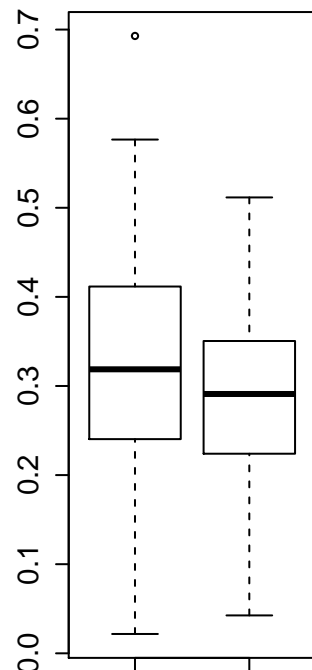**2011**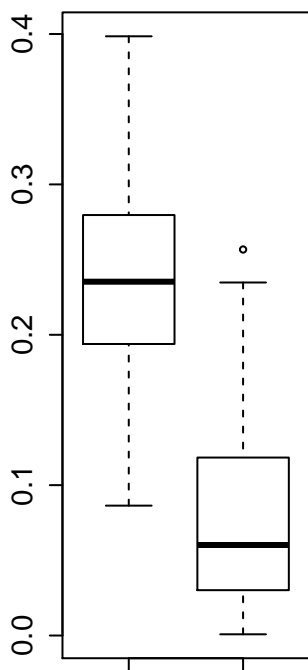**2012**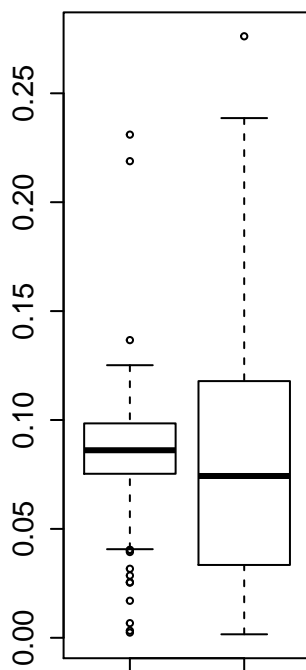**2013**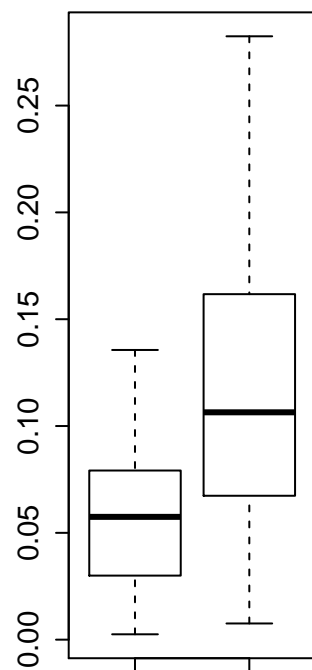

Supplement: Additional file 1: Fig. S1-3. — Plots of the full (left box in each panel) and reduced (right box in each panel) simulations and their predictive distances (y axis). The predictive distance is the difference between the simulated values and the real value from the network. S1 is for mean path length, S2 for degree correlation and S3 for clustering coefficient. See Methods (in the main text) for details on how these were calculated and see Results for which comparisons are statistically significant. (ZIP 21 kb) [file 12862_2016_726_MOESM1_ESM.zip › SN across years figS2 DC.pdf]

**2006**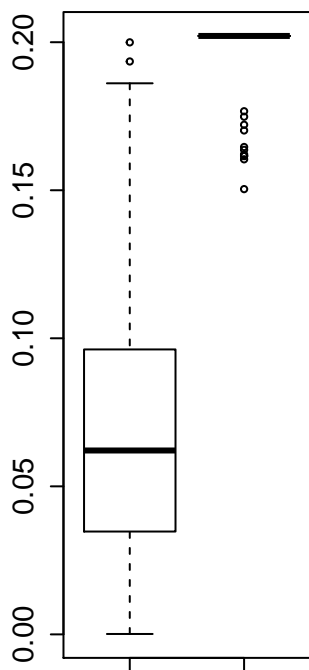**2007**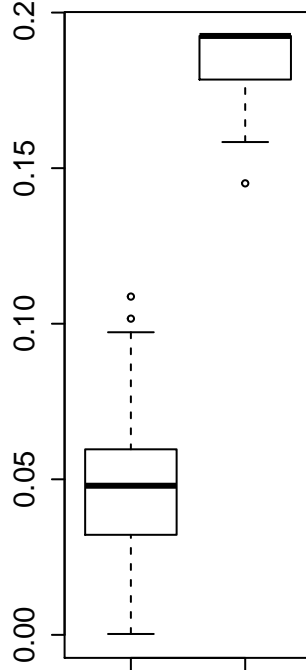**2008**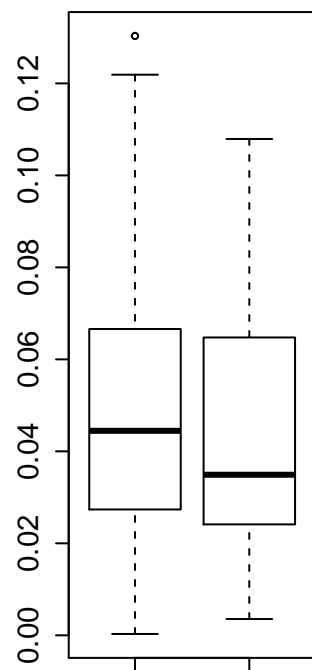**2011**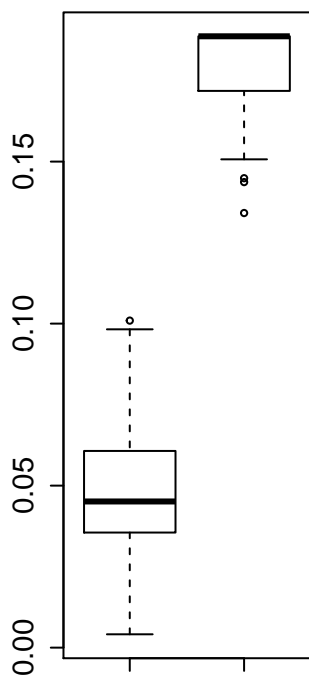**2012**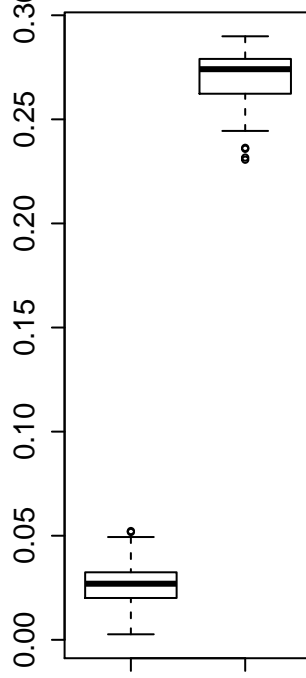**2013**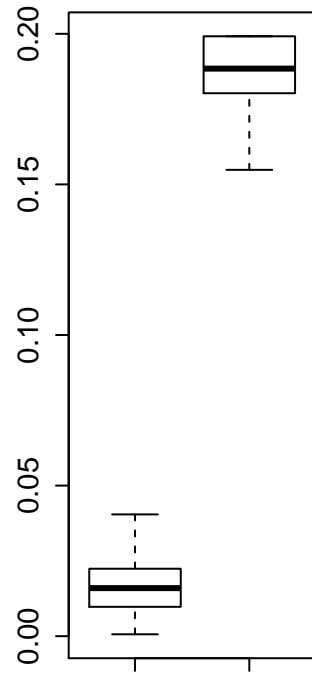

Supplement: Additional file 1: Fig. S1-3. — Plots of the full (left box in each panel) and reduced (right box in each panel) simulations and their predictive distances (y axis). The predictive distance is the difference between the simulated values and the real value from the network. S1 is for mean path length, S2 for degree correlation and S3 for clustering coefficient. See Methods (in the main text) for details on how these were calculated and see Results for which comparisons are statistically significant. (ZIP 21 kb) [file 12862_2016_726_MOESM1_ESM.zip › SN across years figS3 CC.pdf]
