# Supplementary material for: Wild cricket social networks show stability across generations
Source: BMC Evol Biol. 2016 Jul 27;16:151. doi: 10.1186/s12862-016-0726-9 (PMC4964091; doi:10.1186/s12862-016-0726-9)
Supplement: Additional file 2: Table S1. — Characteristics of the cricket population and network statistics for each year. Isolated nodes are individuals that were not recorded to mate or fight another individual. Clustering is the ratio of complete triangles, where three crickets all interact with each other, to all possible triangles, including those where only two of the three crickets actually interact. Betweenness is the number of shortest paths between each pair of crickets in the network that pass through a particular cricket. We have taken the mean across all individuals, giving one measure of how well connected different parts of the network are (high values indicate poorly connected). Path length is the number of edges one must move along to connect any two nodes in a network. We have taken the mean, giving one value for how densely connected the network is (high values indicate low connectivity). (DOCX 16 kb) [file 12862_2016_726_MOESM2_ESM.docx]

## Tables

| Year | Females | Males | | Population | | Sex ratio | | Fights | Mates | |  | | Total interactions | | Isolated nodes | |
| --- | --- | --- | --- | --- | --- | --- | --- | --- | --- | --- | --- | --- | --- | --- | --- | --- |
| 2006 | 82 | 79 | | 161 | | 0.49 | | 127 | 561 | |  | | 688 | | 73 | |
| 2007 | 113 | 95 | | 208 | | 0.46 | | 267 | 917 | |  | | 1184 | | 43 | |
| 2008 | 47 | 32 | | 79 | | 0.41 | | 33 | 248 | |  | | 281 | | 8 | |
| 2011 | 106 | 92 | | 198 | | 0.47 | | 333 | 660 | |  | | 993 | | 61 | |
| 2012 | 69 | 41 | | 110 | | 0.37 | | 156 | 555 | |  | | 711 | | 22 | |
| 2013 | 118 | 121 | | 239 | | 0.51 | | 623 | 1255 | |  | | 1878 | | 42 | |
|  | **Mean burrows visited** | **Mean life span** | **Clustering** | | **Mean Betweenness** | | **Mean path length** | | | **Mean degree** | |  | | **Degree correlation** | | **Density** |
| 2006 | 4.81 | 17.04 | 0.20 | | 52.90 | | 3.08 | | | 2.32 | |  | | -0.02 | | 0.03 |
| 2007 | 6.62 | 24.58 | 0.19 | | 169.50 | | 3.43 | | | 4.01 | |  | | 0.05 | | 0.04 |
| 2008 | 3.80 | 28.59 | 0.13 | | 52.70 | | 3.07 | | | 2.75 | |  | | -0.33 | | 0.11 |
| 2011 | 6.44 | 25.15 | 0.19 | | 132.95 | | 3.29 | | | 3.78 | |  | | -0.03 | | 0.04 |
| 2012 | 4.85 | 26.19 | 0.29 | | 101.50 | | 3.89 | | | 2.86 | |  | | 0.02 | | 0.06 |
| 2013 | 6.05 | 27.87 | 0.21 | | 257.80 | | 4.02 | | | 4.24 | |  | | 0.16 | | 0.04 |

Table S1. Characteristics of the cricket population and network statistics for each year. Isolated nodes are individuals that were not recorded to mate or fight another individual. Clustering is the ratio of complete triangles, where three crickets all interact with each other, to all possible triangles, including those where only two of the three crickets actually interact. Betweenness is the number of shortest paths between each pair of crickets in the network that pass through a particular cricket. We have taken the mean across all individuals, giving one measure of how well connected different parts of the network are (high values indicate poorly connected). Path length is the number of edges one must move along to connect any two nodes in a network. We have taken the mean, giving one value for how densely connected the network is (high values indicate low connectivity).

Mean degree is the average number of unique nodes a node is connected to. Degree correlation is the correlation between the degrees of two nodes at either end of an edge. Density is the proportion of all possible links that actually exist in the network
